# Integrating research and surveillance to mitigate zoonotic disease risk in indigenous communities: Insights from an expert group meeting in Amazonas, Peru

**DOI:** 10.1371/journal.pntd.0014564

**Published:** 2026-07-31

**Authors:** Jocelyn G. Pérez, Rita Ribeiro, Laura Bergner, Nardus Mollentze, Pershing Bustamante-Chauca, Lizandro Gonzales, Roberto C. Rivera, Miguel Bernal, Victor L. Osorio, Sergio Recuenco, Bruno M. Ghersi, Paulo Colchao-Claux, Luis Soldán-Villarreal, William Bardales, Jesús Rodríguez-Chávez, Jorge L. Maicelo Quintana, Carlos Tello, Rafael Tapia-Limonchi, Daniel G. Streicker, Stella M. Chenet

**Affiliations:** 1 School of Biodiversity, One Health and Veterinary Medicine, College of Medical, Veterinary and Life Sciences, University of Glasgow, Glasgow, United Kingdom; 2 Medical Research Council – University of Glasgow Centre for Virus Research, Glasgow, United Kingdom; 3 School of Veterinary Medicine & Biosciences, SRUC, Craibstone, Aberdeen, United Kingdom; 4 Dirección Regional de Salud de Amazonas, Chachapoyas, Perú; 5 Laboratorio Referencial Regional de Salud Pública Amazonas, Dirección Regional de Salud de Amazonas, Chachapoyas, Perú; 6 Red de Salud Condorcanqui, Santa María de Nieva, Amazonas, Perú; 7 Departamento de Medicina Preventiva y Salud Pública, Facultad de Medicina, Universidad Nacional Mayor de San Marcos, Lima, Perú; 8 Tufts University, Medford, Massachusetts, United States of America; 9 Wildlife Conservation Society, Lima, Perú; 10 Yunkawasi, Lima, Perú; 11 Instituto de Investigación en Ganadería y Biotecnología, Facultad de Ingeniería Zootecnista, Agronegocios y Biotecnología, Universidad Nacional Toribio Rodríguez de Mendoza de Amazonas, Chachapoyas, Perú; 12 Servicio Nacional de Sanidad Agraria, Cajamarca, Perú; 13 Instituto de Investigación para el Desarrollo Sustentable de Ceja de Selva, Universidad Nacional Toribio Rodríguez de Mendoza, Chachapoyas, Amazonas, Perú; 14 Instituto de Investigación en Enfermedades Tropicales, Universidad Nacional Toribio Rodríguez de Mendoza de Amazonas, Chachapoyas, Perú; 15 Facultad de Medicina, Universidad Nacional Toribio Rodríguez de Mendoza de Amazonas, Chachapoyas, Perú; University of Massachusetts Amherst, UNITED STATES OF AMERICA

## Introduction

Diseases transmitted from animals to humans disproportionately impact populations living in poverty in tropical and subtropical regions but remain overlooked in research and policy [1]. Given the complex, multi-species biology of these pathogens, neglected zoonotic diseases (NZDs) require a One Health perspective for surveillance and control [2,3]. Monitoring and managing NZDs is particularly challenging in remote indigenous communities, which often suffer regionally disproportionate health disparities [4,5]. Importantly, and in contrast to voluntary isolated or uncontacted communities, these communities are exposed to health threats from the wider society in which they live. Remote indigenous communities may also experience relatively high levels of contact with wildlife through housing practices and animal consumption, including groups that harbor zoonotic pathogens such as bats and rodents [6–9]. Geographical isolation and poverty further limit access to disease prevention and post-exposure healthcare [10,11]. Lack of access to safe water and sanitation may further contribute to NZD spread in these communities [10,11].

**Fig 1:**
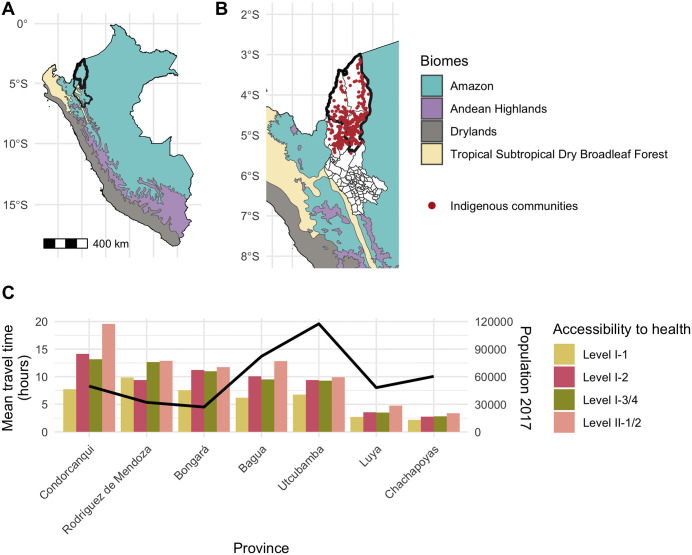
Location of indigenous communities in Condorcanqui and health accessibility. **(A)** Amazonas department is bordered by a dashed black line, and Condorcanqui is bordered by a continuous black line. **(B)** A zoomed-in view showing the districts in Amazonas, Condorcanqui, and the locations of the indigenous communities. Locations of indigenous communities derived from [12]. Biome definitions are based on data from [13,14]. Administrative boundary definitions derived from GADM (https://gadm.org/license.html) and the Peruvian National Open Data Platform (https://www.datosabiertos.gob.pe/dataset/limites-departamentales). **(C)** Mean travel time (hours) to each healthcare level and population size in each province, highlighting poor accessibility to almost all healthcare levels in Condorcanqui, based on [15,16]. The categories of health care centers are: Level I-1 (health posts with health workers not including physicians), Level I-2 (health posts with health workers including physicians), Level I-3 (health centers), Level I-4 (health centers that have ward beds), Level II-1/2 (hospital and clinics).

In May 2023 we held a scoping meeting at Universidad Nacional Toribio Rodríguez de Mendoza in Chachapoyas (Amazonas, Peru). Participants ranged from Peruvian governmental bodies responsible for human and animal health, academics studying human and animal health and ecology, and non-governmental organization concerned with wildlife health and conservation, and sustainable development of communities. The epidemiological situation and disease control measures were presented by health authorities, followed up by a round-table discussion to identify key challenges, and research priorities on NZDs affecting indigenous communities. We focused on the Condorcanqui province in the administrative region of Amazonas, Peru (Fig 1A), which typifies the challenges to NZD management outlined above.

This high-jungle region contains many remote communities of native Amazonian ethnic groups such as Awajún or Wampis, which have distinctive native languages, cultural practices and beliefs. Condorcanqui is also a hotspot of NZDs in Latin America, with a recurring history of human rabies outbreaks transmitted by bats [7] and continuous risks of yellow fever and other infectious diseases (see below). After reviewing the epidemiological situation in the broader region, we identified current barriers to policy change and research. We next developed a set of recommendations for studying and addressing healthcare inequalities that will be relevant to improve NZD surveillance and response in remote indigenous communities more broadly.

## Epidemiological situation

Condorcanqui contains 69% of the indigenous population of Amazonas (total indigenous population: 72,436) and 68% (234/344) of its indigenous communities (Fig 1B) [12]. Many communities are reachable only by river, limiting access to healthcare (Fig 1C).

Data on disease prevalence—where available—are based mostly on passive surveillance or active surveillance following perceived epidemiological shifts [17].

**Fig 2:**
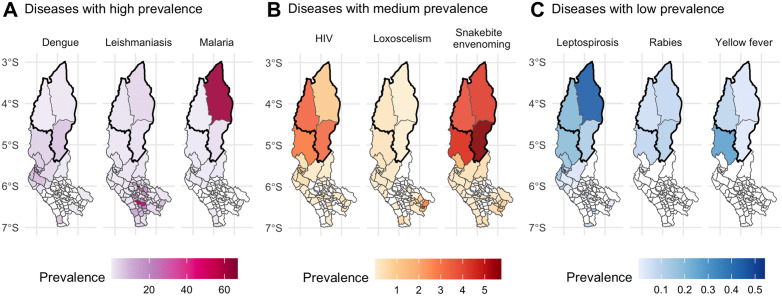
Prevalence of NTDs and other major infectious diseases per district in Amazonas, Peru, from 2000 to 2023. Diseases were grouped by prevalence levels: high **(A)**, medium **(B)** and low **(C)**. Prevalence is expressed as a percentage of cases per total population in 2017, per district [23]. Condorcanqui is bordered in black. Surveillance data supplied by the Centro Nacional de Epidemiología Prevención y Control de Enfermedades - Ministerio de Salud (CDC-MINSA), Peru. Administrative boundary definitions derived from the Peruvian National Open Data Platform (https://www.datosabiertos.gob.pe/dataset/limites-departamentales).

Research studies quantifying prevalence in this region are rare (particularly in indigenous communities), focused on single diseases, and limited to relatively short time periods [18,19]. Meeting participants identified nine infectious and non-infectious diseases of major concern to the region, most of which were neglected tropical diseases (NTDs) (Fig 2). Three of these (rabies, leishmaniasis, leptospirosis) are primarily zoonotic, and two are primarily human-transmitted but also occur in zoonotic/sylvatic cycles (yellow fever, dengue). Two additional infectious diseases (HIV and malaria) are not NTDs but exert considerable direct health burden and have indirect effects on the detection and clinical outcomes of rarer zoonoses [20–22].

Compared to other areas of Amazonas, most of these diseases are more common in Condorcanqui (Fig 2). Furthermore, it is notable that all NTDs highlighted are relatively well-known; other NTDs or NZDs might be present but not subjected to similar diagnostic efforts.

**Fig 3:**
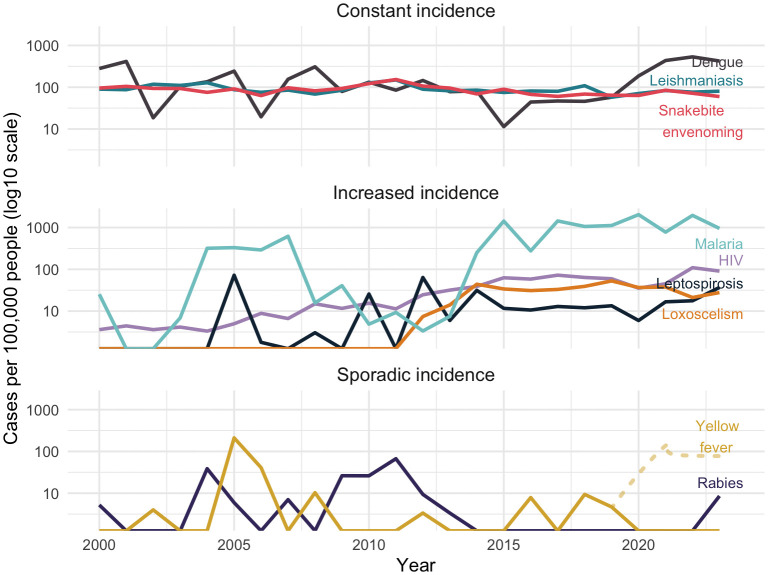
Incidence of NTDs and other major infectious diseases in Amazonas, Peru, from 2000 to 2023. To improve readability, diseases were grouped by overall temporal trend. Incidence is expressed as the number of cases per 100,000 people in 2017.

Among the focal NTDs, dengue, snakebite envenoming and leishmaniasis showed constant incidence over time while rabies and yellow fever were sporadic threats (Fig 3).

The incidence of malaria, HIV, leptospirosis and loxoscelism increased over time, with malaria and HIV exhibiting particularly large increases. It is unclear whether increased incidence reflects improvements in awareness and detection of these diseases or changes in epidemiology (e.g., malaria driven by climate factors [18]).

Condorcanqui is a high-risk area for the emergence and spread of NTDs considering the different factors (e.g., limited accessibility of healthcare, impacts of climate and land use change, poor quality housing and sanitation, cultural beliefs, human activities) that contribute to the observed prevalence of NTDs.

## Challenges and strategies

The challenges to research, diagnostics and treatment of NZDs within indigenous communities identified by our expert panel, along with strategies to resolve them, span both broad-scale policy issues and challenges specific to the research required to inform policy (Table 1). We use Amazonas as an example, but many concepts will be relevant beyond this region.

**Table 1:** Policy and research challenges identified and solutions proposed.

Theme	Challenges	Recommendations
Structural challenges	Compartmentalization of human, domestic animal and wildlife health surveillance programs.	Expand recent approaches to coordinate surveillance and notification system between human, animal, and environmental health entities for decision making using digital platforms (e.g., [24]
	Introduction of zoonotic disease from unanticipated locations.	Accelerate negotiations towards coordinated surveillance across regional and national borders among nations sharing the Amazon basin [25].
Resource allocation	Limited resources for surveillance and control of zoonotic diseases.	Public funding should be evidence-based, incorporating both disease burden (for endemic diseases) and outbreak/emergence risk (for non-endemic diseases).
	Limited healthcare infrastructure, workforce, and diagnostic capacity leads to underestimation of zoonotic disease burden.	Reference laboratories should be established closer to places with high risk of zoonotic disease. (e.g., repurposing of research laboratories from Universidad Nacional Toribio Rodríguez de Mendoza in Chachapoyas during the COVID-19 pandemic). Additional investment required to expand healthcare infrastructure in underserved communities.
Infrastructure & logistics	Low accessibility introduces high transport costs and time loss.	Coordinate trips with existing research programs. If none exist, projects should build local research capacity as a key aim.
	Maintaining cold chain and sample integrity.	Use sample storage options which are not reliant on cold chains. Collaborate with local health posts which have storage facilities available.
Community engagement	Cultural challenges due to beliefs around causes of infectious disease leads to a reluctance to participate in sample collection, treatment or vaccination [7]. Reluctance to engage with outsiders.	Establish previous contact before traveling to communities, integrating local authorities. Obtain printed permission following the national regulations for protocols. Build a multidisciplinary team including sociologists or anthropologists known to communities, local interpreters/guides, and healthcare workers, to minimize risks to new researchers. Obtain informed consent from community chiefs as well as individual participants. Community engagement throughout the lifespan of research projects to strengthen trust. Co-develop educational tools with community health workers on other prevalent diseases, going beyond the scope of individual research projects. Promote the participation of community members that can result in tangible benefits (e.g., hiring local community members for research activities). Communicate research findings, following established best-practices for health research involving marginalized communities [26]. Share samples between public health surveillance and research projects. Coordinate visits among projects to minimize disruption.
	Role of wild animals in both zoonotic disease transmission and broader ecology poorly understood by community members.	Develop community outreach tools for reducing zoonotic disease risk and strategies to protect wild animal species (e.g., pamphlets about zoonotic disease prevention, educational messages about the important ecological niches filled by wild animals). Train hunters to collect samples from wild animals hunted for subsistence (e.g., through a participatory conservation program in a community-based wildlife management [6,9]).
Comorbidities & Under-reporting	High rates of comorbidities such as HIV/AIDS and parasitic diseases that compromise the immune system can influence the likelihood of zoonotic infection [20].	Consider community priorities and health needs beyond zoonotic disease when developing research agendas. Incorporate data on comorbidities into studies of zoonotic disease.
	Lack of surveillance and under-reporting of disease in remote communities.	Improve healthcare infrastructure and build local capacity for diagnostics, unify surveillance efforts across local human and animal health services.

### Policy challenges

There are strategic lines of actions from PAHO and WHO that provide valuable guidance for national health authorities to establish policies to address the health threats at the human-animal-environment interface [2]. However, the following policy challenges are currently faced by the indigenous communities in Peru. Novel zoonotic outbreaks are increasing globally. As occurred during the COVID-19 pandemic, mitigating actions are commonly taken after disease has emerged and spread in humans. To minimize the impact of future pandemics, the implementation of both primary prevention actions (e.g., pathogen discovery and monitoring) and preparedness actions seeking to arrest early transmission chains (e.g., vaccine development) are necessary.

Surveillance underpins both primary prevention and preparedness, enabling actions aiming to prevent spillover (e.g., managing wildlife trade and land use change), rapidly identify the identity and origins of novel human pathogens, and develop diagnostics, therapeutics and vaccines. However, zoonotic surveillance systems—and the primary healthcare systems required to respond to zoonotic threats at the point of emergence—face structural challenges related to sustainability and allocation of funding. A timely and effective zoonotic disease surveillance system can help public health decision makers to set priorities and plan interventions. However, a policy challenge in Peru and elsewhere is the compartmentalization of diagnostic systems for veterinary and human cases of the same diseases. We suggest integrating data across monitoring agencies in a unified resource. Digital platforms of zoonotic disease monitoring at the regional or national level would be valuable for response and preparedness plans if the ethical and legal constraints of data sharing are adequately addressed. Ideally, this approach should integrate human, animal and environmental health data (i.e., a One Health approach). Data sharing in an open platform is also needed to address concerns around the introduction of zoonoses across international borders. Current efforts in Peru such as the avian influenza surveillance system launched in 2023, which integrates data from agencies responsible for agricultural health, agrarian development, wildlife and forestry, and protected areas (Servicio Nacional de Sanidad Agraria [SENASA], Programa de Desarrollo Productivo Agrario Rural [AGRO RURAL], Servicio Nacional Forestal y de Fauna Silvestre [SERFOR], and Servicio Nacional de Áreas Naturales Protegidas por el Estado [SERNANP]) demonstrates the feasibility and value of such an approach [24].

An important challenge in controlling NZDs in remote communities is the lack of access to high-quality health services. For instance, hospital services are concentrated in urban areas of Amazonas (Fig 1C), while people in rural areas lack timely access to healthcare, resulting in limited availability of diagnosis, treatment options, and patient follow-up. Such disparities are further exacerbated by the fragmented Peruvian healthcare system since access to health services depends on the insurance scheme [27]. The lack of adequate and opportune health care in Condorcanqui communities was evident during the COVID-19 pandemic, with a higher incidence rate in indigenous communities compared to other areas in the region [5]. Strengthening primary health care (infrastructure, internet, multidisciplinary teams) is therefore urgently needed. Similarly, there is limited access to veterinary services; more veterinarians are needed locally to improve animal health and to monitor disease emergence.

Localized capacity was deemed critical to address recurring issues of delayed diagnosis and underreporting. Although diagnostic tests for some viral pathogens (e.g., Dengue, COVID-19, Monkeypox, Influenza A and B, Chikungunya, Zika) are currently carried out by local reference laboratories, these laboratories are located in larger cities in Amazonas (Chachapoyas, Bagua). Alternatively, samples may be shipped to the National Institute of Health (Instituto Nacional de Salud; INS) in Lima for confirmatory testing, delaying diagnosis. This could be addressed through strengthening capacity in local reference and research laboratories—for instance, cooperation between these entities improved understanding of risk factors for infection and outcome during the COVID-19 pandemic in Amazonas [19].

Both detection and treatment of NZDs could be improved by re-evaluating investment priorities, ensuring that surveillance and healthcare spending match the risk of high-impact zoonoses and the exposure risk of communities (Figs 1 and 2). The current health system prioritizes a few high-burden diseases (e.g., malaria, dengue). While it is undoubtedly important to direct resources towards these diseases, investment in sustained surveillance, diagnostics and treatment of NZDs could help alleviate the burden of known pathogens (e.g., rabies) and allow identification of as yet unknown pathogens before they become widespread threats [28].

### Research challenges

Determining community needs is necessary to plan research activities that could meet their health agenda. Community health workers identified under-reporting of infectious diseases as a key regional challenge. In Peru more generally, it is necessary that all facilities access the internet and have electronic medical records to ensure up-to-date consensus metadata for monitoring health changes in indigenous populations [27]. The disease prevalence data presented here were collected passively, providing relatively limited insight into the true disease burden on indigenous populations. Enhanced active surveillance, integrating animal and human data including syndromic cases, could help the early detection of spillover events or disease reemergence for developing control strategies. This is particularly important in light of the presence of febrile illnesses such as malaria and chronic immunosuppressive diseases such as HIV in geographically isolated regions of Amazonas (Fig 2), which may mask or exacerbate the impact of NZDs, respectively. Relatively common diseases in the region that initially present with nonspecific febrile illness (malaria, dengue, leptospirosis, yellow fever; Fig 2) may mask the occurrence of other zoonoses given limited diagnostic capacity in the region. The high prevalence of HIV in this region, combined with a lack of specialized care required to prevent progression to AIDS, may further exacerbate the burden of zoonotic NTDs [20].

The remoteness of indigenous communities creates challenges for research attempting to measure the true burden of NTDs and their underlying causes (Table 1, Box 1). We suggest better coordination between academia, external partner institutions and governmental surveillance efforts, to build local capacity and reduce the high costs associated with reaching affected communities. While the importance of research coordination and expanding local capacity as part of global health research programmes is increasingly recognized [29], sustained support at the governmental level is also required. This could be accomplished via networking events and meetings between researchers and the government officials and developing joint training or education programmes to obtain a higher degree or specialize in specific public health topics.

A recurring theme in our discussions was the reluctance of communities to engage with research. Some of these challenges stem from cultural beliefs around the causes of zoonotic diseases or suspicions of outsiders (Table 1). In addition to coordinating visits as suggested above, wider sharing of already collected samples—subject to obtaining informed consent for future use of samples—could help minimize participation fatigue. Further, community engagement throughout the lifespan of a project and beyond, promoting the direct involvement of community members in research activities, and clear communication of results would help to build trust. Crucially, such activities would need explicit support from research funders, and—given the clear ethical and sustainability implications—should be viewed as an integral requirement of research activities involving historically neglected communities.

**Box 1. Challenges to mitigate the risk of a widespread zoonosis in indigenous communities—Rabies as a case study**

**Fig 4:**
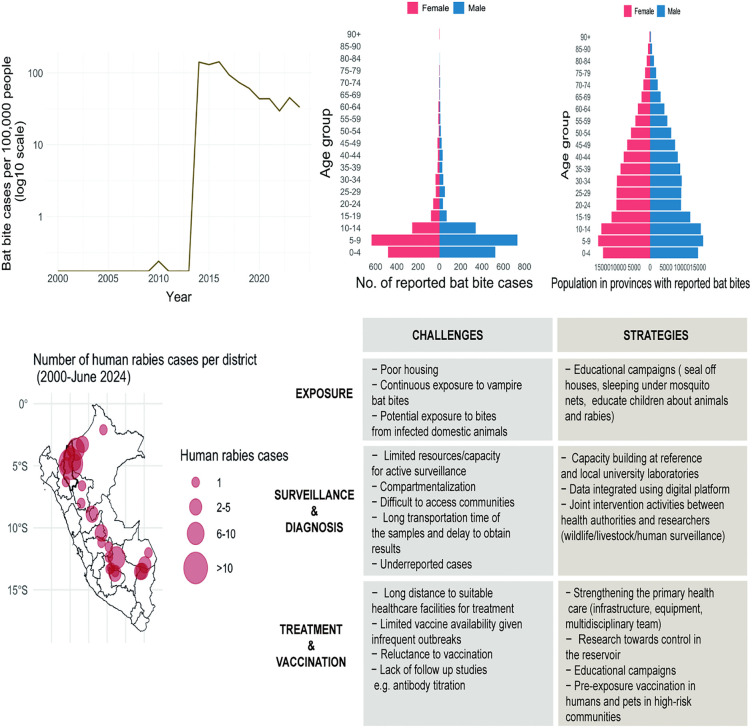
Challenges and strategies to control rabies in indigenous communities in Amazonas. Figures show the bat bite incidence from 2000 to June 2024 in Amazonas, the number of cases by age group, and a population pyramid of the five provinces with reported bat bites (Bagua, Bongara, Condorcanqui, Rodriguez de Mendoza and Utcubamba). Map shows the confirmed human rabies cases in Peru per district from 2000 to June 2024, Amazonas is bordered in black. Surveillance data supplied by the CDC-MINSA and DIRESA-Amazonas. Population data derived from [32]. Administrative boundary definitions derived from GADM (https://gadm.org/license.html).

In Peru, 26.1% of human rabies cases transmitted by vampire bats from 2000 to June 2024 have been reported in Amazonas and 100% of these outbreaks occurred in indigenous communities (Fig 4). During this period, there were 3576 reported bat bite cases in this region, disproportionately affecting children 0–11 years old (Fig 4). Challenges to prevent human rabies in remote Amazonian communities include:

- Chronic exposure to bats due to open-air dwellings, infrequent use of mosquito netting to prevent bat bites, and shifting of bat blood feeding behavior from wildlife to humans or livestock [7].
- Diagnostic results can be delayed due to the long transportation route of samples to the reference laboratory in Bagua and later confirmation at INS-Lima.
- Pre-exposure vaccination campaigns have been implemented in high risk areas [17], but both coverage and duration of immunity is uncertain due to the lack of follow-up antibody titration.
- Reluctance to vaccination due to local beliefs regarding the vaccine or disease etiology is also challenging for health workers, who sometimes face hostility [7]. Educational campaigns would be beneficial in explaining the value of preventing exposure to bat bites and building trust in vaccination.

The Regional Directorate of Health (DIRESA) in Amazonas included human bat-bites as a reportable incident in their surveillance system in 2000, the notification improved progressively and more recent data shows that a considerable problem was missed entirely because there was not a system in place to detect it. This data could help to know the frequency of communities that experience bat bites and identify ecological factors that drive bite and subsequently rabies risk, information that could ultimately guide the distribution of resources, including human vaccines. Joint efforts between health authorities and researchers may aid to fill the gaps of the knowledge of rabies in humans and animals contributing to estimate human local risk [30], and the development of strategies to control rabies outbreaks in livestock and bat populations (e.g., through vaccination [31]).

## Conclusions

Remote indigenous communities are particularly vulnerable to NZDs due to both high human-wildlife contact and lack of access to healthcare. Many communities also face compounding challenges including climate change, incursion by non-indigenous people onto indigenous lands, and development policies that prioritize resource extraction [33]. While development might lead to some positive outcomes (e.g., increased access to healthcare), extractive development models involving land degradation can facilitate disease spread, resulting in higher local disease burdens [33].

In addition to the key policy and research strategies outlined in Table 1, we stress the importance of a One Health approach in both research studies and proposed solutions [3,7]. This would have mutual benefits for researchers, healthcare officials and affected communities. For instance, a One Health approach to managing NZDs might include (1) determining potential pathways of wildlife-human contact, (2) coordinated surveillance in animals and humans, (3) community campaigns to enhance awareness of potential routes of exposure, (4) developing strategies to minimize exposure risks, and (5) building local research capacity to ensure research continuity. Across all stages, seeking the input of communities themselves (i.e., co-development) is critical to ensure proposed research agendas and solutions are embraced.

In summary, our meeting has brought together experts from diverse fields to establish a new network aiming to coordinate research into NZDs in remote indigenous communities in the Amazonas region. Here, we provide guidance to researchers to engage in meaningful collaborations with indigenous communities. Forming research networks to work collaboratively on topics such as NZDs can benefit both individual participants and the wider research community. Developments since this meeting include joint research grant applications, establishing collaborative projects, international online seminars for government officials, and initial planning towards a follow-up meeting in 2026.

## References

[1] World Health Organization. The control of neglected zoonotic diseases: a route to poverty alleviation. Meslin FX, editor. Report of a Joint WHO/DFID-AHP Meeting with the participation of FAO and OIE. Geneva: World Health Organization; 2006. Available from: https://www.who.int/publications/i/item/9789241594301

[2] 59th Directing Council, 73rd Session of the Regional Committee of WHO for the Americas. Pan American Health Organization (PAHO). 2021. Available from: https://www.paho.org/sites/default/files/2021-08/CD59-9-e-one-health.pdf

[3] Webster JP, Gower CM, Knowles SCL, Molyneux DH, Fenton A. One Health - an ecological and evolutionary framework for tackling Neglected Zoonotic Diseases. Evol Appl. 2016;9(2):313–33. doi: 10.1111/eva.12341 26834828 PMC4721077

[4] Kuang-Yao Pan W, Erlien C, Bilsborrow RE. Morbidity and mortality disparities among colonist and indigenous populations in the Ecuadorian Amazon. Soc Sci Med. 2010;70(3):401–11. doi: 10.1016/j.socscimed.2009.09.021 19906478 PMC2814897

[5] Pajuelo-Reyes C, Valencia HJ, Montenegro CC, Quezada E, Gonzales L, Cruz N. Epidemiological analysis of COVID-19 cases in native Amazonian communities from Peru. Epidemiologia. 2021;2(4):4. doi: 10.3390/epidemiologia2040034 36417212 PMC9620947

[6] Pérez JG, Carrera JP, Serrano E, Pittí Y, Maguiña JL, Mentaberre G. Serologic evidence of zoonotic alphaviruses in humans from an indigenous community in the Peruvian Amazon. Am J Trop Med Hyg. 2019;101(6):1212–8. doi: 10.4269/ajtmh.18-0850 31571566 PMC6896884

[7] Stoner-Duncan B, Streicker DG, Tedeschi CM. Vampire bats and rabies: toward an ecological solution to a public health problem. PLoS Negl Trop Dis. 2014;8(6):e2867. doi: 10.1371/journal.pntd.0002867 PMC4063729 24945360

[8] Vivi-Oliveira VK, Junior AAP, Lacerda TEJ, Rozental T, Lemos ERSde, Espinosa MM, et al. Serological evidence of Bartonellosis in an indigenous community in the Brazilian Legal Amazonia. Zoonoses Public Health. 2021;68(8):987–92. doi: 10.1111/zph.12881 34259394

[9] Menajovsky MF, Espunyes J, Ulloa G, Calderon M, Diestra A, Malaga E. Toxoplasma gondii in a remote subsistence hunting-based indigenous community of the Peruvian Amazon. Trop Med Infect Dis. 2024;9(5):5. doi: 10.3390/tropicalmed9050098 PMC11125861 38787031

[10] Slimming PAT, Orellana ER, Maynas JS. Structural determinants of indigenous health: a photovoice study in the Peruvian Amazon. AlterNative Int J Indigenous Peoples. 2014;10(2):123–33. doi: 10.1177/117718011401000203

[11] United Nations. State of the world’s indigenous peoples: indigenous peoples’ access to health services. New York: United Nations; 2016. doi: 10.18356/7914b045-en

[12] Instituto del Bien Común. Sistema de Información sobre Comunidades Nativas de la Amazonía Peruana (SICNA). 2024. [cited 2024 Aug 9]. Available from: https://ibcperu.org/servicios/sicna/

[13] Olson DM, Dinerstein E, Wikramanayake ED, Burgess ND, Powell GVN, Underwood EC, et al. Terrestrial ecoregions of the world: a new map of life on earth: a new global map of terrestrial ecoregions provides an innovative tool for conserving biodiversity. BioScience. 2001;51(11):11. doi: 10.1641/0006-3568(2001)051[0933:TEOTWA]2.0.CO;2

[14] Polk MH, Mishra NB, Young KR, Mainali K. Greening and Browning Trends across Peru’s Diverse Environments. Remote Sensing. 2020;12(15):2418. doi: 10.3390/rs12152418

[15] Weiss DJ, Nelson A, Gibson HS, Temperley W, Peedell S, Lieber A, et al. A global map of travel time to cities to assess inequalities in accessibility in 2015. Nature. 2018;553(7688):333–6. doi: 10.1038/nature25181 29320477

[16] Superintendencia Nacional de Salud. Registro Nacional de IPRESS - RENIPRESS. Available from: https://www.gob.pe/susalud

[17] Recuenco SE. Rabies vaccines, prophylactic, Peru: Massive rabies pre-exposure prophylaxis for high-risk populations. In: Ertl HCJ, editor. Rabies and rabies vaccines. Cham: Springer International Publishing; 2020. p. 83–101. doi: 10.1007/978-3-030-21084-7_5

[18] Saavedra-Samillán M, Burgos F, García Huamán F, Valdivia HO, Gamboa D, Chenet SM. Spatiotemporal dynamics of malaria and climate influence on its incidence in Condorcanqui Province, 2005–2022. Malar J. 2024;23(1):1. doi: 10.1186/s12936-024-05193-6 39695645 PMC11657927

[19] Campos CJ, Pajuelo-Reyes C, Rojas LM, De La Cruz-Vargas JA, Tejedo JR, Tapia-Limonchi R, et al. Prevalence of SARS-CoV-2 variants and disease outcome of COVID-19 patients in the Amazonas region of Peru. Am J Trop Med Hyg. 2023;109(3):523–6. doi: 10.4269/ajtmh.22-0739 37524331 PMC10484257

[20] Laiton-Donato K, Ávila-Robayo P, Páez-Martinez A, Benjumea-Nieto P, Usme-Ciro JA, Pinzón-Nariño N, et al. Progressive vaccinia acquired through zoonotic transmission in a patient with HIV/AIDS, Colombia. Emerg Infect Dis. 2020;26(3):601–5. doi: 10.3201/eid2603.191365 32091366 PMC7045850

[21] Cota GF, Sousa MRde, Mendonça ALPde, Patrocinio A, Assunção LS, Faria SRde. Leishmania-HIV Co-infection: clinical presentation and outcomes in an urban area in Brazil. PLoS Negl Trop Dis. 2014;8(4):e2816. doi: 10.1371/journal.pntd.0002816 PMC3990491 24743472

[22] Halliday JEB, Carugati M, Snavely ME, Allan KJ, Beamesderfer J, Ladbury GAF, et al. Zoonotic causes of febrile illness in malaria endemic countries: a systematic review. Lancet Infect Dis. 2020;20(2):e27–37. doi: 10.1016/S1473-3099(19)30629-2 32006517 PMC7212085

[23] Centro Nacional de Seguimiento y Evaluación (CEPLAN). Información para el planeamiento a nivel departamental, provincial y distrital. 2020. [cited 2024 Dec 24]. Available from: https://www.ceplan.gob.pe/informacion-sobre-zonas-y-departamentos-del-peru/

[24] Guía para la vigilancia y respuesta integrada de influenza tipo A altamente patógena en Perú. Lima, Perú: Ministerio de Salud, Ministerio de Desarrollo Agrario y Riego, and Ministerio del Ambiente; 2024. Available from: https://www.dge.gob.pe/sala-influenza-aviar/docs/guia-integrada-firmada-influenza-tipo-a.pdf

[25] Declaration of Belém. The Amazon Cooperation Treaty. 2023. Available from: https://otca.org/en/wp-content/uploads/2023/10/Declaration-of-Belem.pdf

[26] Lin CY, Loyola-Sanchez A, Boyling E, Barnabe C. Community engagement approaches for Indigenous health research: recommendations based on an integrative review. BMJ Open. 2020;10(11):e039736. doi: 10.1136/bmjopen-2020-039736 33247010 PMC7703446

[27] Carrillo-Larco RM, Guzman-Vilca WC, Leon-Velarde F, Bernabe-Ortiz A, Jimenez MM, Penny ME, et al. Peru - progress in health and sciences in 200 years of independence. Lancet Reg Health Am. 2021;7:100148. doi: 10.1016/j.lana.2021.100148 36777656 PMC9904031

[28] Halliday J, Daborn C, Auty H, Mtema Z, Lembo T, Bronsvoort BMD, et al. Bringing together emerging and endemic zoonoses surveillance: shared challenges and a common solution. Philos Trans R Soc Lond B Biol Sci. 2012;367(1604):2872–80. doi: 10.1098/rstb.2011.0362 22966142 PMC3427560

[29] Kelly TR, Machalaba C, Karesh WB, Crook PZ, Gilardi K, Nziza J, et al. Implementing One Health approaches to confront emerging and re-emerging zoonotic disease threats: lessons from PREDICT. One Health Outlook. 2020;2:1. doi: 10.1186/s42522-019-0007-9 33824944 PMC7149069

[30] Brock Fenton M, Streicker DG, Racey PA, Tuttle MD, Medellin RA, Daley MJ, et al. Knowledge gaps about rabies transmission from vampire bats to humans. Nat Ecol Evol. 2020;4(4):517–8. doi: 10.1038/s41559-020-1144-3 32203471 PMC7896415

[31] Cárdenas-Canales EM, Velasco-Villa A, Ellison JA, Satheshkumar PS, Osorio JE, Rocke TE. A recombinant rabies vaccine that prevents viral shedding in rabid common vampire bats (Desmodus rotundus). PLoS Negl Trop Dis. 2022;16(8):e0010699. doi: 10.1371/journal.pntd.0010699 36026522 PMC9455887

[32] Instituto Nacional de Estadística e Informática. Censos Nacionales 2017: XII de Población, VII de Vivienda Y III de Comunidades Indígenas [Internet]. 2025 [cited 2025 Feb 16]. Available from: https://censos2017.inei.gob.pe/redatam/

[33] Castro MC, Baeza A, Codeço CT, Cucunubá ZM, Dal’Asta AP, De Leo GA, et al. Development, environmental degradation, and disease spread in the Brazilian Amazon. PLoS Biol. 2019;17(11):e3000526. doi: 10.1371/journal.pbio.3000526 31730640 PMC6881077

